# Impact of Lymphadenectomy on the Oncologic Outcome of Patients with Adrenocortical Carcinoma—A Systematic Review and Meta-Analysis

**DOI:** 10.3390/cancers14020291

**Published:** 2022-01-07

**Authors:** Anne Hendricks, Sophie Müller, Martin Fassnacht, Christoph-Thomas Germer, Verena A. Wiegering, Armin Wiegering, Joachim Reibetanz

**Affiliations:** 1 Department of General, Visceral, Transplant, Vascular and Pediatric Surgery, University Hospital Wuerzburg, Oberduerrbacher Strasse 6, 97080 Wuerzburg, Germany; Hendricks_A@ukw.de (A.H.); mueller_s27@ukw.de (S.M.); germer_c@ukw.de (C.-T.G.); Wiegering_A@ukw.de (A.W.); 2 Department of Internal Medicine I, Division of Endocrinology and Diabetology, University Hospital Wuerzburg, Oberduerrbacher Strasse 6, 97080 Wuerzburg, Germany; fassnacht_m@ukw.de; 3 Department of Pediatric Hematology, Oncology and Stem Cell Transplantation, University Children’s Hospital Wuerzburg, Josef-Schneider-Strasse 2, 97080 Wuerzburg, Germany; Wiegering_V@ukw.de

**Keywords:** adrenocortical carcinoma, adrenal cancer, lymphadenectomy, lymph node dissection, LND, LNE, review, meta-analysis

## Abstract

**Simple Summary:**

The concept of locoregional lymphadenectomy (LND) in adrenocortical cancer (ACC) has gained interest in recent years, but its definite prognostic and therapeutic significance remains to be established. We undertook a systematic review and meta-analysis to determine the oncologic value of LND in ACC, focusing on overall survival as the primary endpoint. Eleven studies were identified and five were included in the meta-analysis, all of them were retrospective studies. Three studies reported the impact of LND on disease-specific survival in patients with stage I–III ACC and revealed a survival benefit of LND, whereas studies including patients with stage I–IV ACC (*N* = 2) did not show a survival benefit of LND. In summary, our results demonstrate an oncologic benefit of LND in patients undergoing curative-intended surgery for at least localized ACC (stage I–III).

**Abstract:**

(1) Background: Locoregional lymphadenectomy (LND) in adrenocortical carcinoma (ACC) may impact oncological outcome, but the findings from individual studies are conflicting. The aim of this systematic review and meta-analysis was to determine the oncological value of LND in ACC by summarizing the available literature. (2) Methods: A systematic search on studies published until December 2020 was performed according to the PRISMA statement. The primary outcome was the impact of lymphadenectomy on overall survival (OS). Two separate meta-analyses were performed for studies including patients with localized ACC (stage I–III) and those including all tumor stages (I–IV). Secondary endpoints included postoperative mortality and length of hospital stay (LOS). (3) Results: 11 publications were identified for inclusion. All studies were retrospective studies, published between 2001–2020, and 5 were included in the meta-analysis. Three studies (*N* = 807 patients) reported the impact of LND on disease-specific survival in patients with stage I–III ACC and revealed a survival benefit of LND (hazard ratio (HR) = 0.42, 95% confidence interval (95% CI): 0.26–0.68). Based on results of studies including patients with ACC stage I–IV (2 studies, *N* = 3934 patients), LND was not associated with a survival benefit (HR = 1.00, 95% CI: 0.70–1.42). None of the included studies showed an association between LND and postoperative mortality or LOS. (4) Conclusion: Locoregional lymphadenectomy seems to offer an oncologic benefit in patients undergoing curative-intended surgery for localized ACC (stage I–III).

## 1. Introduction

Adrenocortical carcinoma (ACC) is a rare disease with a stage-dependent but generally poor prognosis mainly due to a high recurrence rate after treatment [1,2,3,4,5]. Despite continual improvements in the standardization of systemic treatments [3,6,7,8,9] complete surgical resection of the primary tumor is the mainstay in non-metastatic disease and is the only treatment that offers the prospect of cure [4,10,11,12]. Besides tumor stage and tumor grade, lymph node involvement was shown to be an important negative prognostic factor [13,14,15].

In recent years, the concept of lymphadenectomy (LND) in ACC has gained greater interest and in cases of suspected or proven ACC, LND during primary surgery now appears to be a guideline-compliant procedure [7,8,16]. However, published results regarding LND in ACC are conflicting [17,18,19,20,21,22,23,24,25,26,27], so its prognostic and therapeutic significance remains to be established. Further, published series usually include a limited number of patients and the reported lymphadenectomy rate within such series is low [4,18,21,23]. Consequently, it is difficult to draw conclusions on the role of LND in ACC based on current information. We therefore undertook a systematic review and subsequent meta-analysis to summarize and analyze available literature to determine the oncological value of LND in ACC.

## 2. Materials and Methods

### 2.1. Study Design and Search Strategy

**Figure 1:**
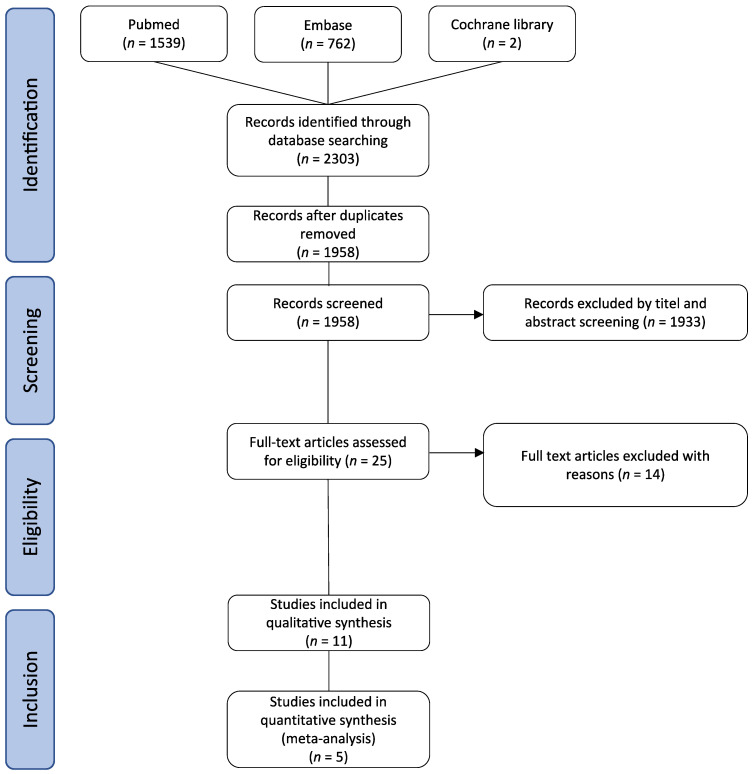
Flow chart of the literature search and selection process.

This study is a systematic review and meta-analysis performed according to PRISMA (Preferred Reporting Items for Systematic Reviews and Meta-Analyses) guidelines [28]. We registered our systematic review on Prospero (registration ID is 301757). A systematic literature search of PubMed, EMBASE and Cochrane Library electronic databases was performed for articles published until December 2020. The search terminus was “adrenal [Title/Abstract] OR adrenocortical* [Title/Abstract] AND cancer [Title/Abstract] OR malign* [Title/Abstract] OR carcinom* [Title/Abstract] AND lymphnode* [Title/Abstract] OR lymphadenctom* [Title/Abstract] OR lymph node dissection [Title/Abstract] OR LNE [Title/Abstract]”. All relevant articles, irrespective of year of publication, type of publication, or publication status, were included in the search. The reference lists of studies of interest were manually reviewed for additional articles. Three reviewers (A.H., S.M., J.R.) independently screened titles and abstracts of all publications identified by this database search. The remaining studies were reviewed in full text to determine their eligibility for inclusion and analysis. Discordant judgment was resolved by discussion and consensus. The selection process is presented in the PRISMA flowchart (Figure 1). If two separate studies analyzed data from the same database and during overlapping time periods, one study was selected according to the below-mentioned criteria and included. In cases of incomplete data/information, the principal author was contacted and provided the missing data/information when possible.

### 2.2. Study Selection, Inclusion Criteria and Outcome Measures

To be included in the current meta-analysis, eligible studies had to fulfill the following criteria: (1) surgery (open or laparoscopic) for histologically confirmed ACC in non-pediatric patients (>14 years), (2) the surgery was a primary procedure (no recurrent disease), (3) providing results on the oncological value of lymphadenectomy versus no lymphadenectomy (LND versus NoLND) during primary tumor resection, (4) report of at least one of the outcomes of interest. The primary outcome was the impact of lymphadenectomy on overall survival (OS). Secondary endpoints included the duration of postoperative hospital stay and postoperative mortality. Two separate meta-analyses were performed for studies including only patients with stage I–III cancers (localized disease) [17,20,24] and those also including advanced or metastatic disease (stage IV) [25,27]. The definition of LND and NoLND was left to the respective study and ranged from the examination of a predefined number of lymph nodes (e.g., ≥5) to “surgeon’s effort to perform a lymphadenectomy” to not being specifically defined. Open and laparoscopic procedures were included. Studies which did not report sufficient data (e.g., reviews and case reports) were excluded from the analysis. Further, studies investigating ACC in children ≤14 years of age, pregnant women, recurrent disease, adrenal tumors other than ACC, and metastases in the adrenal gland were excluded. All relevant texts, tables and figures of the included studies were reviewed for data extraction.

### 2.3. Assessment of Risk of Bias

To assess the risk of bias, the ROBINS-I (risk of bias in non-randomized studies of interventions) tool was applied. The included studies were rated in the categories low, moderate, serious and critical risk of bias [29]. The assessment determined an adequate result for all studies included in our analysis (Table A1).

### 2.4. Statistical Analysis

Review Manager 5 was used to analyze data and to generate forest plots for data presentation. Studies were analyzed using generic inverse variance meta-analysis with a random effects model. The measures of effects hazard ratio (HR) with corresponding 95% confidence intervals (95% CI) were calculated. Statistical heterogeneity was assessed using the Chi2 test of heterogeneity and I2 statistic.

## 3. Results

### 3.1. Study Characteristics

The literature search identified 2303 studies. After removing duplicates, 1953 records were screened by titles and abstracts, resulting in 25 potentially relevant articles. Of these, 14 studies were excluded due to missing data pertaining to our predefined endpoints. Eleven studies were included in the qualitative analysis [17,18,19,20,21,22,23,24,25,26,27]. These studies reported patients from five different cancer databases, and study characteristics are summarized in Table A2. For meta-analysis, only one study from each database was selected, that (1) addressed the question of LND in ACC in the context of the current study most accurately, (2) provided sufficient data for analysis, (3) provided the most precise patient selection (see inclusion criteria), and/or (4) covered the longest inclusion period. Lastly, (5) studies published between 2012 and 2018 were included in the quantitative analysis [17,20,24,25,27].

### 3.2. Lymphadenectomy and Survival

To analyze the impact of lymphadenectomy on overall survival, we performed two separate meta-analyses for studies including only patients with stage I–III ACC and studies including all tumor stages I–IV. Because advanced and metastatic ACC per se drive worse survival, a separate analysis of the subgroups seemed justified. 

**Figure 2:**
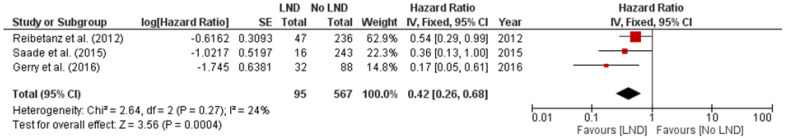
Overall survival after lymphadenectomy (LND) vs. no lymphadenectomy (NoLND) for patients with ACC stage I–III. Of the 662 patients who received surgery in total, 95 patients underwent LND while for 567 patients NoLND was performed. The size of the red square indicates the size (power) of the respective study, the black diamond below the studies represents the overall effect.

The studies of Gerry et al., Saade et al. and Reibetanz et al. reported the impact of LND on disease specific survival in patients with stage I–III ACC [17,20,24]. In total, 807 patients with stage I–III ACC were included of which 662 received surgery. Ninety-five patients underwent LND while 567 did not. Based on these studies, patients with LND showed a survival benefit (hazard ratio (HR) = 0.42, 95% confidence interval (95% CI): 0.26–0.68; *p* = 0.0004) (Figure 2). The test for heterogeneity showed a low value with I^2^ 24%.

**Figure 3:**
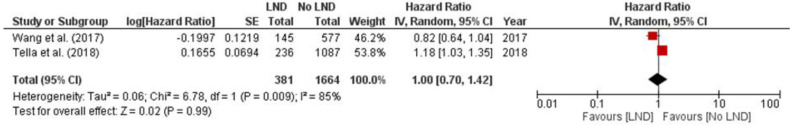
Overall survival after lymphadenectomy (LND) vs. no lymphadenectomy (NoLND) for patients with ACC stage I–IV. Of the 2025 patients who received surgery in total, 381 patients underwent LND, while for 1664 patients NoLND was performed. The size of the red square indicates the size (power) of the respective study, the black diamond below the studies represents the overall effect.

We further analyzed two studies reporting the impact of lymphadenectomy in patients with stage I–IV ACC [25,27]. These studies included 3934 patients of which 2025 received surgery. In total, 381 patients underwent LND, while 1664 patients did not. Based on these studies, LND was not associated with a survival benefit (HR = 1.00, 95% CI: 0.70–1.42; *p* = 0.99) (Figure 3). The test for heterogeneity showed a high heterogeneity with I^2^ 85%.

In a subgroup analysis, Tella et al. analyzed stage I–III and stage IV separately [25]. No survival benefit was shown for patients with LND in stage I–III. For stage IV disease, a significant longer overall survival (OS) was shown in the LND group compared to the NoLND group (15 vs. 6 months, *p* < 0.001). The study by Tran et al. reported similar findings [26]. In a query of the SEER database, they identified 320 patients with stage III and IV disease (patients with distant metastasis were excluded). In total, 280 patients received surgery of whom 83 patients had LND. They found that LND was associated with improved cancer specific survival in patients with T4 tumors.

### 3.3. Postoperative Mortality

Three studies reported the postoperative mortality for the LND and NoLND group [17,19,20]. These studies used different time points as the cut-off for mortality, which makes a direct comparison difficult. Deschner et al. reported a 90-days-mortality of 3 (2.0%) in LND and 25 (3.3%) in NoLND patients [19]. Reibetanz et al. evaluated the 30-days-mortality and found 0 for LND and 3 (1.3%) for NoLND patients [17]. Gerry et al. only reported the in-hospital mortality. There were no in-hospital deaths for LND and 2 (1.3%) for NoLND patients [20]. These findings showed no significant difference of postoperative mortality in both groups.

### 3.4. Length of Hospital Stay

**Figure 4:**
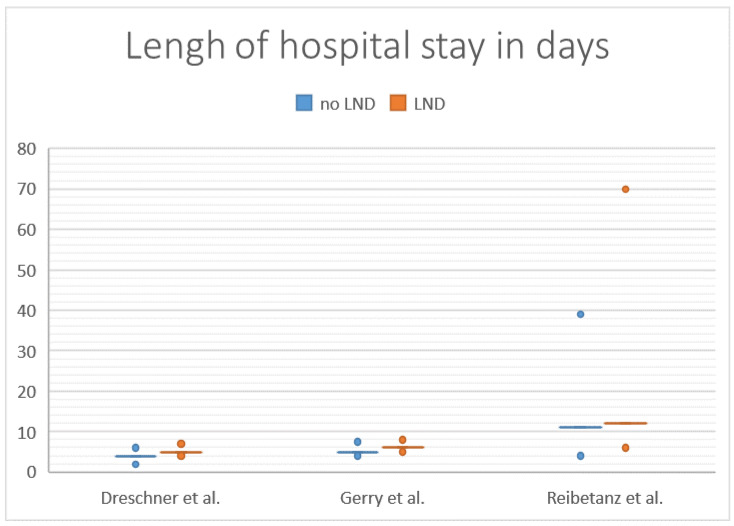
Length of hospital stay (days) of patients undergoing lymphadenectomy (LND) versus no lymphadenectomy (no LND).

Three studies compared the length of hospital stay (LOS) for patients with LND and NoLND [17,19,20]. Gerry et al. reported a median LOS of 5 days (4–7.5) for patients with LND and 6 days for patients with NoLND (range: 5–8) [20] Deschner et al. reported the median LOS for LND patients of 4 days (range: 2–6) and for NoLND patients of 6 days (5–8) [19]. Reibetanz et al. found a median LOS of 11 days (range: 4–39) for patients with LND and 12 days (range: 6–70) for NoLND patients, and therefore twice as long compared to other studies [17]. None of the studies showed a significant impact of LND on the LOS (Figure 4).

## 4. Discussion

To our knowledge, this is the first meta-analysis assessing the therapeutic benefit of LND in ACC. Of the 11 identified studies, 5 were eligible for meta-analysis regarding the survival difference of LND versus NoLND. The joint evaluation of the three included studies suggests an oncologic benefit of LND in patients undergoing curative-intended surgery for localized ACC (stage I–III). On the other hand, LND had no impact on survival when patients with advanced or metastatic ACC were included in the analysis (stage I–IV). However, subgroup analysis of some studies revealed a benefit of LND even in advanced tumor stages (stage III–IV) [26].

The fact that the observed oncological benefit of LND in stage I–III ACC disappeared if patients with metastasized disease were included in the meta-analysis (Figure 2 and Figure 3) is perhaps to be expected. On the one hand, the indication for surgery per se in metastasized ACC is debatable and is rarely intended to be curative. On the other hand, it is conceivable that at the stage of metastatic disease, a locoregional procedure (additional LND) apparently will not improve prognosis. However, the studies of Tella et al. and Tran et al. reported that even in stage III and IV disease, LND was associated with a survival benefit. These authors propose a reduction of local tumor burden as a possible reason [25,26].

Current literature indicates that lymph node involvement in ACC represents an independent predictor of survival. Panjwani et al. showed that patients with positive lymph nodes have a three-fold increased risk of death, and thus lymph node metastases represent an even more unfavorable prognostic factor than a positive resection margin [13]. Similarly, Deschner et al. demonstrated that the detection of positive lymph nodes (N1) is associated with a significantly worse prognosis compared to negative (or unexamined) lymph nodes (N0, Nx) [19]. Additional studies support these results by identifying lymph node involvement as a negative prognostic factor regarding tumor recurrence, disease-free and disease-specific survival [4,17,18,24,27]. Even in advanced tumor stages (stage III, IV), the presence of lymph node metastases was shown to be associated with worse overall survival [26]. Therefore, the clinical significance of peritumoral lymph nodes in ACC is plausible, and their removal under the objective of systematic lymphadenectomy (during primary surgery) seems sensible. Logically, the necessity of the LND in ACC is now increasingly being recommended by international guidelines [8,16,30,31,32]. Which retroperitoneal compartments might reasonably be cleared in terms of LND during index surgery to reduce the risk of lymph node or locoregional recurrence in ACC has recently been proposed by our group [33]. Accordingly, in right-sided ACC the fatty tissue cranial and caudal to the renal hilum should be excised, as well as the lymph-nodes in the inter-aortocaval space and right of the inferior caval vein. In left-sided ACC, lymph-nodes and soft tissue in the area of the renal hilum and in the para-aortic and interaorto-caval space should be cleared.

Although international guidelines now recommend LND in suspected or proven ACC, this comprehensive review of current literature demonstrates that the role of lymphadenectomy in ACC is still insufficiently defined. Even in large series, the LND rate is very low (5.1–35.3%) when contrasted against the high rate of lymph node involvement (8–55%) [17,18,19,20,21,22,23,24,25,26,27]. For example, Deschner et al. reported that the rate of lymphadenectomy and pathological examination, even in academic centers, is only about 20% [19]. This is remarkable as patients’ lymph node status is a basic component of all previously published staging systems for ACC [1,34,35,36]. Further, recent studies demonstrate a clear relationship between the number of lymph nodes removed and the probability of detecting lymph node metastases [19]. However, the number of examined lymph nodes was not well documented in all previous studies. Moreover, it is not clear if the reported lymph node numbers represent the total number of removed lymph nodes. In the future, a recommendation on the minimal number of pathologically examined lymph nodes would be reasonable.

The role of LND on postoperative hospital stay was reported in three studies [17,19,20]. In these studies, the authors did not identify any difference in the groups of LND and NoLND. Conclusively, LND seems safe and does not cause an increased operative risk.

There are some limitations to this systematic review and meta-analysis. First, we were only able to include retrospective studies in our analysis since, to our knowledge, no prospective studies are available or ongoing. Second, the definitions of lymphadenectomy were heterogeneous and ranged from the examination of a predefined number of lymph nodes (e.g., ≥5) to “surgeon’s effort to perform a lymphadenectomy” to not being defined at all. Therefore, the reported baseline for each individual study is likely different. Third, the groups of LND and NoLND patients varied in size with the vast majority of patients not undergoing LND. Fourth, the included studies differed in the staging system used to stage ACC (ENSAT versus AJCC/UICC), which might have impacted our results. Finally, there are some further studies that reported the impact of LND in ACC but were not included in the meta-analysis due to above mentioned reasons and our predefined inclusion criteria. For example, Alanee et al. and Nilubol et al. analyzed a patient cohort with stage I–IV ACC from the SEER database. They did not find a significant correlation between LND and disease specific survival (94.5 ± 14.8 vs. 121.9 ± 5.6 months, *p* = 0.3) [18,23] Deschner et al. evaluated a cohort of 897 patients from the NCDB with margin-negative resection for ACC and 46 patients underwent LND. Distant metastasis and T4 tumors were excluded. The results again revealed no benefit on survival for LND compared to NoLND (OS: 62 months (95% CI 20.8-not reached) vs. OS: 74.8 months (95% CI 40.1 months-not reached)) [19]. In 2001, Icard et al. performed a workup of the French Association of Endocrine surgeons’ database. A total of 253 surgically treated patients were included in the analysis, 89 of whom underwent LND. It was found that LND did not cause a significant difference in survival [21].

Due to the overall incidence of ACC, we decided not to restrict our search strategy to modern studies, that had been published since the first report regarding the issue of LND in ACC in 2012 [17]. We are aware that—especially in studies of the “pre-LND-era”—on many occasions, lymph nodes were (and still are) removed unintentionally (particularly with the adjacent kidney) and not because the surgeon was looking for lymph nodes. Moreover, perioperative treatment and adjuvant therapy has evolved in recent years, and the overall prognosis in ACC now is only comparable to a limited extend to that of early 2000. Nonetheless, when it comes to the question of lymphadenectomy, we believe that consideration of older studies is also justified in this regard, as the basic surgical technique and implementation of LND (in several malignancies) has probably changed little over time.

Our study has several strengths. To our knowledge, this is the first systematic review and meta-analysis on the issue of LND and survival in ACC patients. Numerous international databases were included which provides a more comprehensive overview of ACC patients compared to single center studies. Further, our study summarizes and evaluates all available data on the impact of LND in ACC. As ACC is a rare disease and no prospective randomized controlled studies are available regarding the issue of LND, this systematic review with meta-analysis provides timely and relevant data for the surgical treatment of ACC.

## 5. Conclusions

In conclusion, our meta-analysis suggests an oncologic benefit of LND in patients undergoing curative-intended surgery for localized ACC (stage I–III). Accordingly, our study supports the recommendation of recently published international guidelines to include LND in the initial surgery in patients with (suspected or proven) localized ACC. However, we are aware that this recommendation is still based on retrospective data only and conclusions should therefore be drawn cautiously.

## Appendix A

**Table A1:** Assessment of risk of bias using the ROBINS-I tool (risk of bias in non-randomized studies of interventions): Low risk of bias = the study is comparable to a well-performed randomized trial; moderate risk of bias = the study appears to provide sound evidence for a non-randomized study but cannot be considered comparable to a well-performed randomized trial; serious risk of bias = the study has some important problems; critical risk of bias = the study is too problematic to provide any useful evidence and should not be included in any synthesis.

Author, Year	Baseline Confounding	Selection of Participants	Classification of Intervention	Deviation From Intended Intervention	Missing Data	Measurement of Outcomes	Selection of Reported Results	Overall Risk of Bias
Alanee, 2015 [18]	Moderate	Low	Low	Low	Low	Low	Low	Moderate
Dreschner, 2020 [19]	Moderate	Low	Moderate	Low	Low	Low	Low	Moderate
Gerry, 2016 [20]	Moderate	Low	Serious	Low	Low	Low	Low	Moderate
Icard, 2001 [21]	Moderate	Low	Serious	Low	Low	Low	Low	Moderate
Marincola, 2018 [22]	Moderate	Low	Serious	Low	Low	Low	Low	Moderate
Nibulol, 2016 [23]	Moderate	Low	Low	Low	Low	Low	Low	Moderate
Reibetanz, 2012 [17]	Moderate	Low	Low	Low	Low	Low	Low	Moderate
Saade, 2015 [24]	Moderate	Low	Low	Low	Low	Low	Low	Moderate
Tella, 2018 [25]	Moderate	Low	Moderate	Low	Low	Low	Low	Moderate
Tran, 2013 [26]	Moderate	Low	Moderate	Low	Low	Low	Low	Moderate
Wang, 2017 [27]	Moderate	Low	Low	Low	Low	Low	Low	Moderate

**Table A2:** Characteristics of included studies. LND = lymphadenectomy, NoLND = no lymphadenectomy, LN-I = lymph node involvement, SEER = Surveillance, Epidemiology and End Results Database of the National Cancer Institute, NCDB = National Cancer Database, ACCG = Adrenocortical Carcinoma Group, ACC = adrenocortical carcinoma, ENSAT = European Network for the Study of Adrenal Tumors, AFCE = French Association of Endocrine Surgeons, nr = not reported, AJCC = American Joint Committee of Cancer.

Author	Year	Country	Database	Data Collection	Population	Staging System Used	Included Tumor Stages	Surgery Performed	LND	noLND	LN-I (%)
Alanee et al. [18]	2015	USA	SEER	1991–2011	1732	Not specifically mentioned	I–IV, metastatic disease included	1037 (59.9%)	56 (5.4%)	981 (94.6%)	30.1%
Dreschner et al. [19]	2020	USA	NCDB	2004–2015	897	Not specifically mentioned	Stage I–III	897 (100%)	46 (5.1%)	851 (94.9%)	16.3%
Gerry et al. [20]	2016	USA	ACCG	1993–2014	265	AJCC	Stage I–III, metastatic disease excluded	120 (45.3%)	32 (26.7%)	88 (73.3%)	8%
Icard et al. [21]	2001	France	AFCE	1978–1997	253	Modified MacFarlane	I–IV, metastatic disease included	252 (99.6%)	89 (35.3%)	163 (64.7%)	nr
Marincola et al. [22]	2018	USA	ACCG	1993–2014	265	AJCC	metastatic disease excluded	158 (59.6%)	37 (23.4%)	121 (76.6%)	9.5%
Nilubol et al. [23]	2015	USA	SEER	1973–2011	1525	‘localized, regional, and distant metastatic disease’	I–IV, metastatic disease included	802 (52.6%)	67 (8.4%)	735 (91.6%)	12.8%
Reibetanz et al. [17]	2012	Germany	German ACC Registry	1981–2009	283	ENSAT	ENSAT I–III	283 (100%)	47 (16.6%)	236 (83.4%)	28%
Saade et al. [24]	2015	USA	SEER	1988–2009	259	ENSAT	ENSAT I–III	259 (100%)	16 (6.2%)	243 (93.8%)	43%
Tella et al. [25]	2018	USA	NCDB	2004–2015	3185	AJCC	Stage I–IV, metastatic disease included	1559 (48.9%) I–III 824 IV 735	I–III 125 (17.9%) IV 111 (17.8%)	I–III 574 (82.1%) IV 513 (82.2%)	55%
Tran et al. [26]	2013	USA	SEER	1988–2009	320	AJCC	AJCC III and IV, distant metastatic disease excluded	280 (87.5%)	83 (29.6%)	237 (84.6%)	35%
Wang et al. [27]	2017	USA	SEER	1973–2014	749	AJCC and ENSAT	I–IV, metastatic disease included	722 (96.4%)	145 (20.1%)	577 (79.9%)	13.7%
